# Microbial diversity in fecal samples depends on DNA extraction method: easyMag DNA extraction compared to QIAamp DNA stool mini kit extraction

**DOI:** 10.1186/1756-0500-7-50

**Published:** 2014-01-21

**Authors:** Hengameh Mirsepasi, Søren Persson, Carsten Struve, Lee O B Andersen, Andreas M Petersen, Karen A Krogfelt

**Affiliations:** 1 Department of Microbiology & Infection Control, Statens Serum Institut, Copenhagen DK-2300 S, Denmark; 2 Department of Gastroenterology, Hvidovre University Hospital, Kettegård Allé 30, Hvidovre DK-2650, Denmark

**Keywords:** Fecal DNA extraction, DNA measurement, Inflammatory bowel disease, Denaturing gradient gel electrophoresis, easyMag®, QIAamp DNA stool mini kit, NanoDrop®, Qubit® system

## Abstract

**Background:**

There are challenges, when extracting bacterial DNA from specimens for molecular diagnostics, since fecal samples also contain DNA from human cells and many different substances derived from food, cell residues and medication that can inhibit downstream PCR. The purpose of the study was to evaluate two different DNA extraction methods in order to choose the most efficient method for studying intestinal bacterial diversity using Denaturing Gradient Gel Electrophoresis (DGGE).

**Findings:**

In this study, a semi-automatic DNA extraction system (easyMag®, BioMérieux, Marcy I’Etoile, France) and a manual one (QIAamp DNA Stool Mini Kit, Qiagen, Hilden, Germany) were tested on stool samples collected from 3 patients with Inflammatory Bowel disease (IBD) and 5 healthy individuals.

DNA extracts obtained by the QIAamp DNA Stool Mini Kit yield a higher amount of DNA compared to DNA extracts obtained by easyMag® from the same fecal samples. Furthermore, DNA extracts obtained using easyMag® seemed to contain inhibitory compounds, since in order to perform a successful PCR-analysis, the sample should be diluted at least 10 times. DGGE performed on PCR from DNA extracted by QIAamp DNA Stool Mini Kit DNA was very successful.

**Conclusion:**

QIAamp DNA Stool Mini Kit DNA extracts are optimal for DGGE runs and this extraction method yields a higher amount of DNA compared to easyMag®.

## Background

The human intestinal microbiota is very complex and varies among individuals. This variation is believed to play a significant role in human health and disease [1,2]. The conventional method used for several decades to identify gastrointestinal bacterial populations has been culturing on selective media followed by biochemical characterization and serotyping, a clear disadvantage when identifying slow-growing or fastidious organisms. Only a small percentage of intestinal microorganisms have been identified by these conventional culture techniques, while recent molecular techniques have provided new knowledge about the complexity of the intestinal micro-flora [3,4]. The first step in molecular diagnostics is the extraction of intact template DNA, which may be generated by boiling bacterial colonies from cultured samples or by DNA extraction directly from the sample. The extraction of fecal DNA is a challenge, since feces not only contains bacterial and human cells but also many different substances derived from for example food, medicine, secondary cell metabolites etc. that can inhibit downstream PCR [5-9]. Various extraction methods have been developed and evaluated [10-13]. These methods are mainly based on chemical and mechanical lysis in the presence of buffers and chelating agents protecting the liberated DNA from degradation, followed by charge-dependent binding to an immobilized matrix that permits washing and elution of pure DNA. When using Denaturing Gradient Gel Electrophoresis (DGGE) of *16S* rDNA it has been demonstrated that the diversity of the microbiota in patients with Inflammatory Bowel Disease (IBD) is less complex than in healthy subjects [14]; nevertheless, the influence of DNA extraction methods is unknown.

In this study, the semi-automated NucliSENS® easyMag system was tested and compared to the manual QIAamp DNA Stool Mini Kit. easyMag® is based on off-board bacterial lysis followed by automated DNA extraction using magnetic beads with bound silica particles. The QIAamp DNA Stool Mini Kit is a manual procedure extracting DNA from chemically and mechanically lysed bacteria on spin columns with bound silica [13,15]. The DNA amount was measured by two different methods. Finally, PCR-DGGE was applied on the DNA extracts from both extraction procedures in order to evaluate the efficiency of the two extraction methods for determining the bacterial diversity in fecal samples from IBD patients and from healthy controls.

## Findings

### Materials and methods

#### Human fecal samples

Fecal samples were obtained from each of three IBD patients and five healthy individuals. Subjects were between 22 and 47 years of age. Each stool sample was split into equal portions (100 mg) and stored at -80°C until processing.

#### DNA extraction by the QIAamp DNA stool MiniKit

DNA extraction was performed according to the instructions of the manufacturer (QIAGEN, Hilden, Germany) with the following modifications: 100 mg fecal sample was mixed with 1.4 mL ASL buffer in a 2 mL tube and vortexed until the sample was thoroughly homogenized. Samples were subsequently mixed with 0.2 g sterile zirconia/silica beads (diameter, 0.1 mm; Biospec Product, ROTH, Karlsruhe, Germany). Hereafter, the samples were processed on a TissueLyser (Qiagen Retsch GmbH, Hannover, Germany) for 6 minutes at 30 Hz [16,17]. Lysis was completed at a temperature of 95°C for 5 minutes. Finally, DNA was extracted according to the instruction of the QIAamp DNA stool MiniKit and eluted in 100 μL elution buffer provided in the kit.

#### DNA extraction by NucliSENS® easyMag

DNA extraction was performed according to the manufacturer’s instructions (NucliSENS**®**.bioMèrieux, France) with some modifications [18,19]. Briefly, 100 mg fecal sample was mixed with 400 μL Lysis Buffer 1 and vortexed using Mylab (Vortex-Mixer SLV-6, Seoulin Bioscience Co., Ltd, Korea) for 10 minutes until the fecal sample was thoroughly homogenized. The samples were subsequently centrifuged for 5 minutes at 13,000 rpm. Hereafter, 140 μL magnetic silica was added to each tube and thoroughly mixed with the sample. The remaining steps of the DNA extraction process were performed by the robot according to protocol A and eluted in 110 μL elution buffer (provided by easyMag**®**) [20].

#### DNA quantification

NanoDrop® (NanoDrop products, Wilmington, DE, USA), and Qubit® (Qubit™ fluorometer, Invitrogen, CA 92008, USA) were used in order to identify the most suitable method for measuring purified DNA from the fecal samples. Nanodrop® measures anything that absorbs light at 260 nm, which could be single-stranded or double-stranded DNA, RNA, proteins or contaminants [21]. The Qubit fluorometer is based on dyes that emit fluorescence when binding to DNA [6,21,22].

#### PCR amplification for denaturing gradient gel electrophoresis

The V2-V3 region of the *16S* rDNA gene was amplified by universal primer set HDA 1 position 338–357: (5′ACT CCT ACG GGA GGC AGC AGT′3) and HDA 2 position 539–561: (5′GTA TTA CCG CGG CTG CTG GCA C–′3) [8]. The forward primer, HDA 1, was at the 5′end labeled with GC clamp (5′CGC CCG GGG CGC GCC CCG GGC GGG GCG GGG GCA CGGGGG G ′3). All primers were purchased from MWG-eurofins, Ebersberg, Germany). PCRs were performed in a total volume of 50 μL containing 20 μL of 5 PRIME Mastermix (MasterMix-100Rxns, 5PRIME GmbH, Hamburg), 0.8 μM primer HDA 1-GC, 0.8 μM primer HDA 2, 10 μL of DNA template (DNA concentrations shown in Table 1) and, finally, 4 μL RNase free water (Qiagen, Hilden, Germany, also used to dilute the DNA extracts in this study). The PCR was performed using the following conditions: preheating at 94°C for 4 minutes proceeded by 30 cycles of denaturing at 94°C for 30 sec, annealing at 56°C for 30 sec, elongation at 68°C for 45 sec, and finally a single step of 68°C for 7 minutes; the PCR products were run on a 0.8% agarose gel.

**Table 1:** Quantification of extracted DNA from fecal samples

**Sample nr.**	**NanoDrop® (μg/mL)**			**Qubit® (μg/mL)**		
	**Qiagen**	**EasyMag 35 μL silica**	**EasyMag 140 μL silica**	**Qiagen**	**EasyMag 35 μL silica**	**EasyMag 140 μL silica**
HC-1	17.5	41.9	13.7	2.9	6.5	0.5
HC-2	40.1	9.6	41.4	6.9	0.7	1.2
HC-3	42.5	52.4	50.8	4.5	1.2	0.7
HC-8	18.5	10.3	36.1	3.4	1.3	0.6
HC-10	73.8	17.4	33.4	28.8	2.5	1.9
IBD-1*	9.5	6.7	32.2	0.6	0.8	1.0
IBD-2*	5.6	3.4	18.4	0.2	1.2	0.3
IBD-3*	55.5	5.9	21.2	2.7	0.4	0.5
Average	32.9	18.5	30.9	6.3	1.8	0.8
Standard deviation	24.1	18.4	12.5	9.4	2.0	0.5

*Inflammatory Bowel Disease (IBD), Healthy controls (HC).

The table shows faecal DNA concentration of extracts quantified using NanoDrop® and Qubit®. DNA extraction by EasyMag® with 35 μL and 140 μl silica yields in average 18.5 (SD 18.4) and 30.9 μg (SD 12.5) DNA, respectively and DNA extraction by Qiagen yields in average 32.9 μg (SD 24.1) DNA, using NanoDrop® instrument. Same faecal DNA extracts measured using Qubit® instrument for DNA by EasyMag® with 35 μL and 140 μL silica, yields a concentration of 1.8 (SD 2.0) and 0.8 μg (SD 0.5) DNA, respectively, while DNA extraction by Qiagen yields a concentration of 6.3 μg/ml (SD 9.4) DNA.

#### Denaturing gradient gel electrophoresis

PCR fragments were separated by DGGE as described by Myuzer [23] with DCode System according to the manufacturer’s instructions (Bio-Rad Laboratories, Hercules, USA). 8% Polyacrylamide (vol/vol) (ratio of acrylamid:bisacrylamide (37.5:1)) were diluted in 0.5xTAE buffer with pH 8.0 using a gradient ranging from 35% to 65% (100% acrylamide corresponds to 7 M urea and 40% (vol/vol) formamide) [17]. Gels were cast using a gradient maker and a pump with a flow speed of 5 mL per minute. After polymerization of the gel (2 hours), a 3% stacking gel without denaturing chemicals was cast, and an appropriate comb was subsequently inserted and left for 30 minutes for polymerization. Gels were run at 60°C for 16 hours at a constant voltage of 70 V in 0.5 × TAE buffer. After electrophoresis, gels were stained with GelRED (Biotium, Denmark) for 45 minutes and analyzed using an ultraviolet trans-illuminator (BIO RAD, Universal HOOD II, Germany).

### Results

The automated easyMag® protocol recommends the use of 140 μL silica [19] to extract fecal DNA. In this study, both 140 and 35 μL silica were tested and the amount of extracted fecal DNA was compared to the amount of extracted fecal DNA using the QIAamp DNA Stool Mini Kit (Qiagen). Both the Qubit® system and NanoDrop® instrument were used to measure the amount of extracted DNA. As shown in Table 1, faecal DNA extraction using easyMag® with 140 and 35 μL silica yielded an average of 30.9 μg/mL (Standard Deviation (SD) 12.5) and 18.5 μg/mL (SD 18.4) DNA, respectively using NanoDrop®, while faecal samples purified using QIAamp DNA Stool Mini Kit (Qiagen) yielded an average of 32.9 μg/mL (SD 24.1) DNA (Table 1). When DNA extracts were measured with the Qubit® system, the average concentration of DNA was measured to be 0.8 μg/mL (SD 0.5), 1.8 μg/mL (SD 2.0) and 6.3 μg/mL (SD 9.4), for easyMag® with 140, 35 μL silica and Qiagen respectively (Table 1).

**Figure 1:**
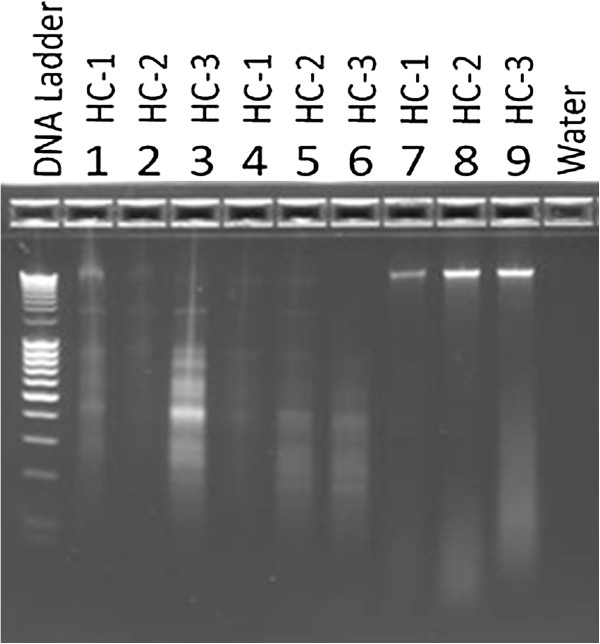
**Electrophoresis gel of 3 healthy individuals DNA extracts: HC-1, HC-2, HC-3, respectively.** Lane 1-3: DNA extracted using easyMag® with 35 μL silica. Lane 4-6: DNA extracted using easyMag® with 140 μL silica. Lane 7–9: DNA extracted using Qiagen method.

DNA extracts from stool specimens HC-1, HC-2, and HC-3 from the healthy individuals were visualized using electrophoresis on a 2% agarose gel. Figure 1, lanes 1–3 and 4–6 representing DNA extracted by easyMag® with 35 and 140 μL silica, respectively, show smears and/or multiple bands, suggesting DNA degradation or fragmentation. The densities of the bands correlate with the quantities of the DNA measured by the Qubit® system, suggesting that higher quality of DNA with less degradation was obtained from samples extracted by Qiagen, which also appears as distinct bands of high molecular weight in lanes 7–9 in Figure 1.

**Figure 2:**
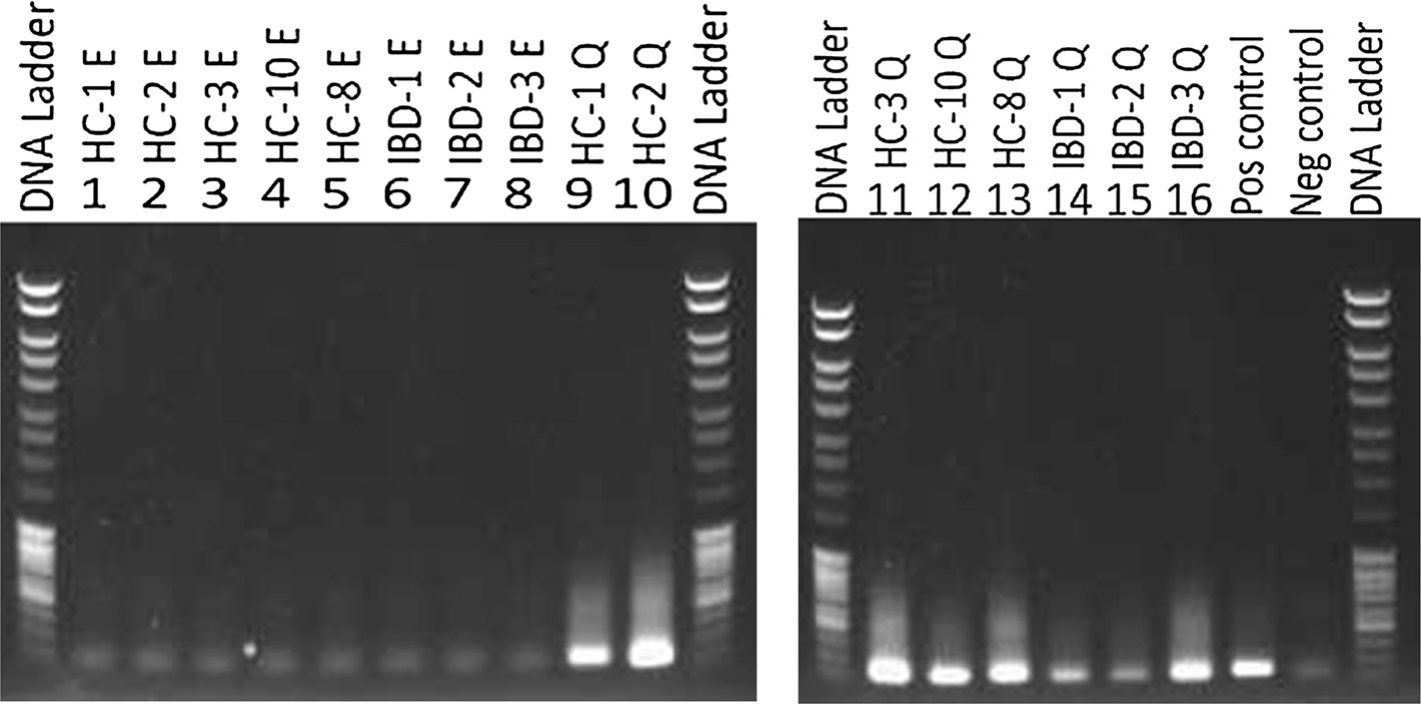
**Gel electrophoresis of 16S rDNA PCR products run on the 0.2% gel. Lane 1-8: PCR products using DNA extracted by easyMag® with 140 μL silica.** Lanes 9-16: PCR products using DNA extracted by Qiagen. Lanes 17-18: positive (*Escherichia coli*) and negative control, respectively.

**Figure 3:**
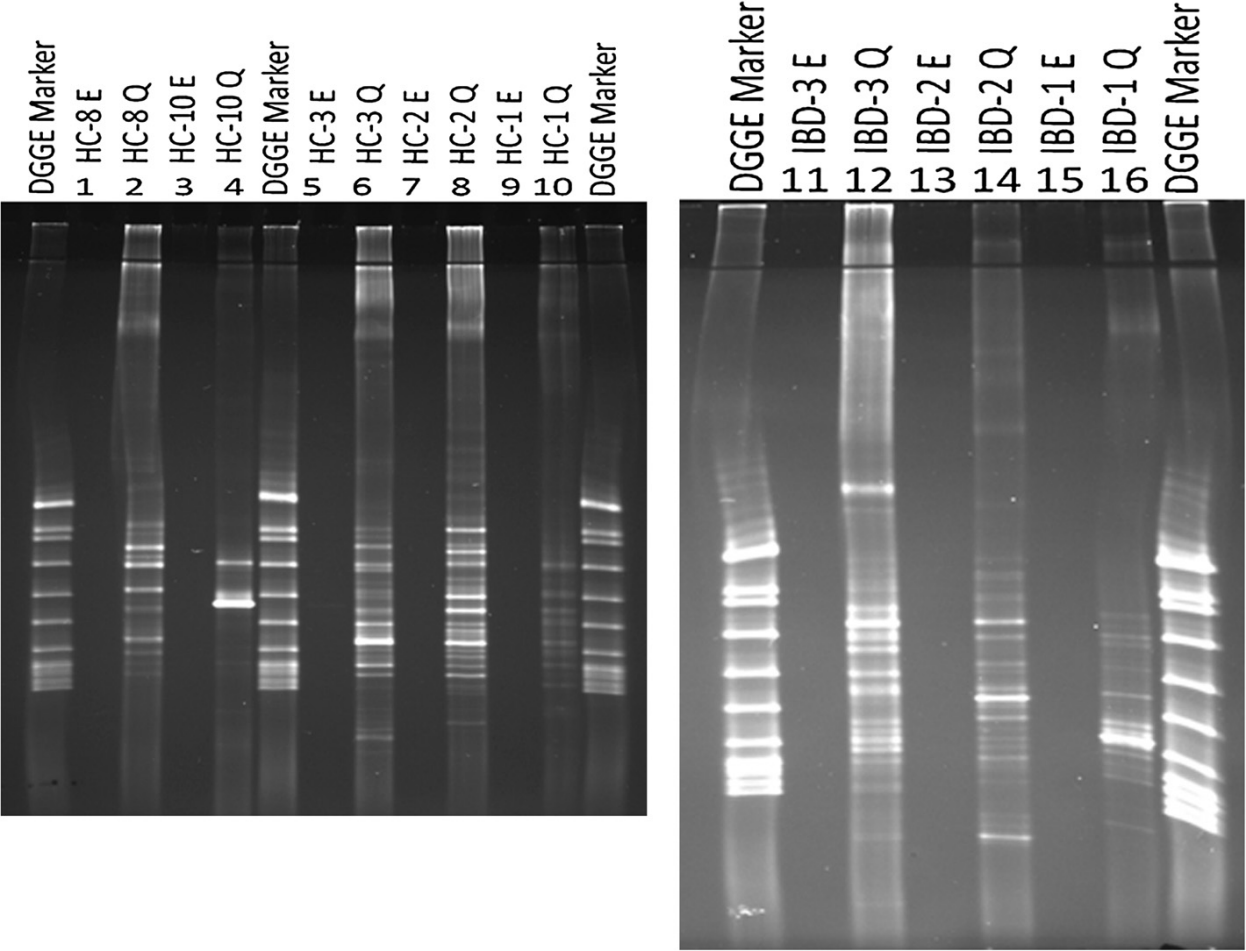
**DGGE gel pictures show, *16S *rDNA PCR products on faecal DNA extracts obtained using easyMag® with140 μL silica and Qiagen methods.** Lanes 1, 3, 5, 7, 9, 11, 13, and 15 are *16S* rDNA PCR products using DNA extracted by easyMag®. Lanes 2, 4, 6, 8, 10, 12, 14, and 16 are *16S* rDNA PCR products using DNA extracted by Qiagen.

Thereafter, *16S* rDNA PCR products were visualized using electrophoresis on 2% agarose gel and analysed by DGGE (Figures 2 and 3). Lanes exhibiting *16S* rDNA PCR products obtained from easyMag® DNA were blank, while PCR products obtained from DNA extracted by Qiagen were clearly visible. This part of the experiment was repeated twice.

**Figure 4:**
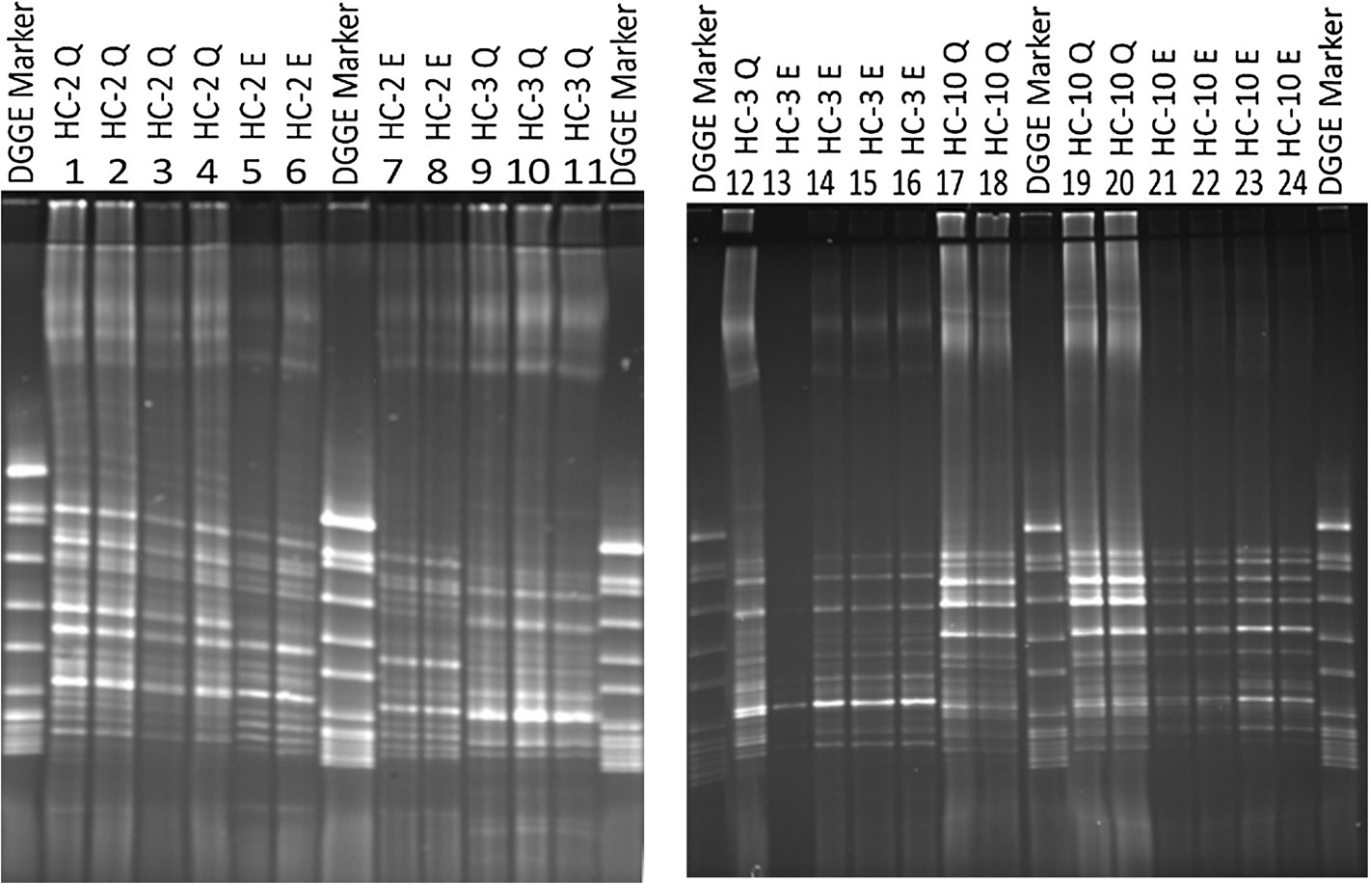
**DGGE gel pictures show *16S *rDNA PCR products of 3 healthy individuals' diluted faecal DNA extracts: 5, 10, 15 and 20 times, respectively.** Lanes 1-4, 9-12, 17-20 show *16S* rDNA PCR products of diluted DNA extracted using Qiagen. Lanes 5-8, 13-16, 21-24 show*16S* rDNA PCR products of diluted DNA extracted using easyMag® with 140 μL silica.

**Figure 5:**
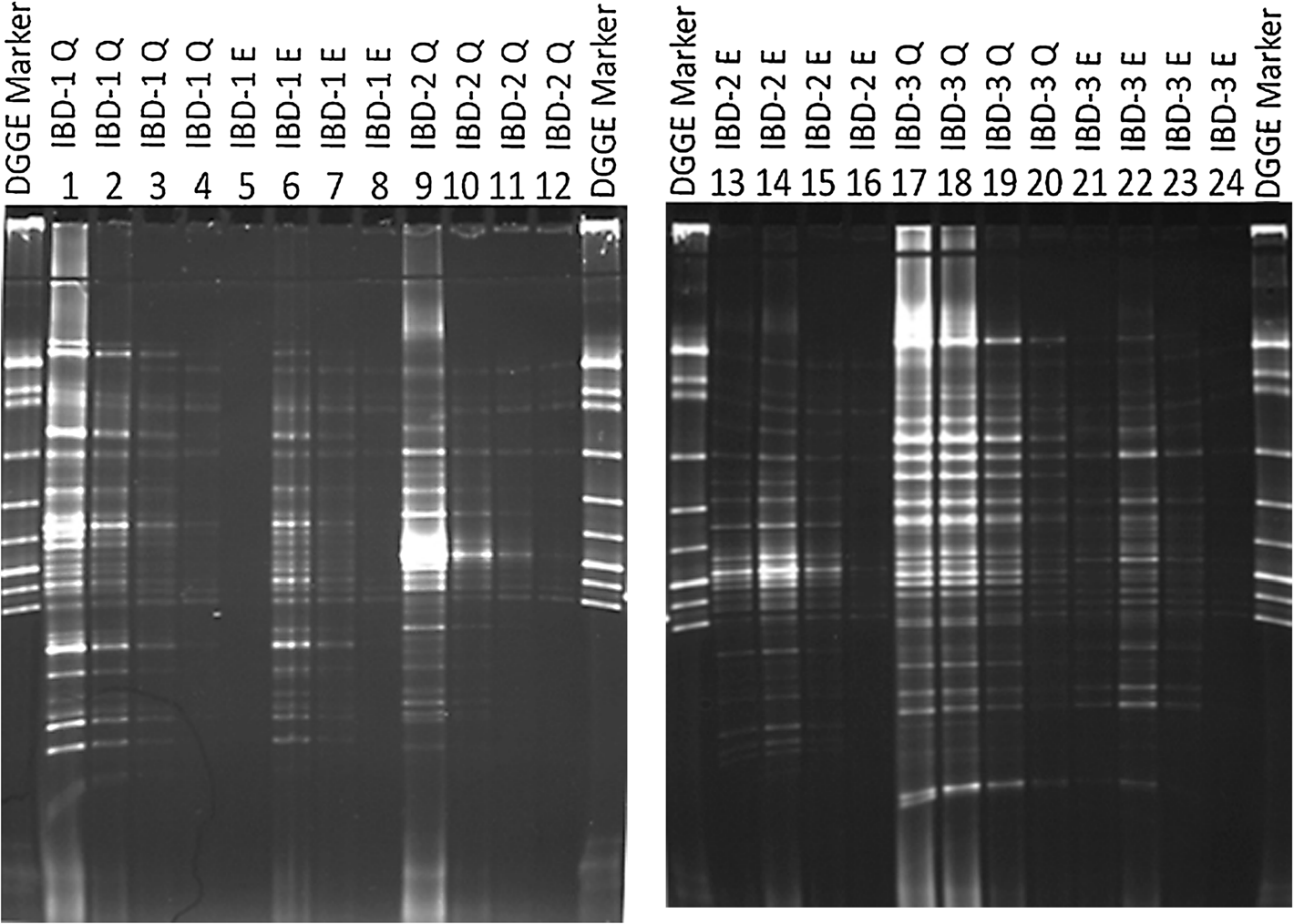
**DGGE gel pictures show *16S *rDNA PCR products of 3 IBD patients' diluted faecal DNA extracts: 5, 10, 15 and 20 times, respectively.** Lanes 1-4, 9-12, 17-20 show 16S rDNA PCR products of diluted DNA extracted using Qiagen. Lanes 5-8, 13-16, 21-24 show *16S* rDNA PCR products of diluted DNA extracted using easyMag® with 140 μL silica.

In order to investigate whether impurities and/or inhibitory compounds had any effect on the visualisation of the DNA, DNA extractions from HC-1, HC-2, HC-3, IBD1, IBD2 and IBD3 were diluted 5, 10, 15, and 20 times. PCR was performed and the PCR-products were analysed by DGGE. As Figure 4 shows, *16S* rDNA PCR products obtained using easyMag® revealed more bands and showed bands with higher densities in the lanes where the DNA was diluted 10 and 15 times. Additionally, almost identical bands appear for each faecal sample in DGGE gel lane in both DNA extracts; diluted easyMag® DNA extracts and Qiagen DNA extracts (Figures 4 and 5). However, the lanes in DGGE gels representing DNA extracted by Qiagen show brightest bands in comparison to DNA extracted by easyMag®. These results were confirmed on diluted DNA extracted by easyMag® of three IBD patients, using DGGE (Figure 5).

### Discussion

In this study the goal was to investigate the DNA extraction quality of two systems, a semi-automatic and a manual DNA extraction system for the purpose of downstream PCR-DGGE analysis. In order to compare these two methods a quantitation of small amounts of DNA is necessary. Based on the results of gel electrophoresis of DNA extracts (Figure 1), there is a greater consistency between the Qubit® system measurement and the density of bands occurring in the gel electrophoresis. Probably NanoDrop® measures other compounds than DNA such as RNA and protein residues with a similar absorption, making this measuring method inconsistent. Differences between DNA amount were tested using One-way Analysis of variance test, which shows significant differences in DNA concentration with no reservation for which measuring method was used (P <0.0001***).

Results of the DNA measurements (Qubit®) and the density of bands in gel electrophoresis showed a relatively low amount of DNA extracted by easyMag® compared to DNA extracted by Qiagen. It was noted that using less silica than suggested by the easyMag® manufacturer resulted in a higher DNA yield. An explanation could be that when using the semi-automatic easyMag® with 140 μL silica relatively more non-DNA compounds such as protein will be extracted from the sample compared to 35 μL silica. These non-DNA compounds probably interfere with the DNA extraction. Diluting the DNA extracts and thereby the non-DNA inhibiting compounds 10 and 15 times resulted in more bands as revealed on DGGE gels. One of the goals of this study was to investigate whether easyMag® can be used to extract small amounts of fecal DNA. IBD patients frequently have a reduced diversity of bacterial DNA in comparison to healthy controls [24-26]. Our results demonstrate that easyMag® can extract DNA from fecal samples and the method is suitable for extracting DNA from fecal samples of IBD patients. However, the DGGE gel lanes representing *16S* rDNA PCR products of the DNA extracted by Qiagen revealed clearly more visible bands in comparison with DGGE gel lanes representing *16S* rDNA PCR products derived from DNA extracted by easyMag®. The reason why Qiagen extracted DNA is of better quality may be found in the method used to extract fecal DNA. The Qiagen DNA extraction method was combined with TissueLyser, which enhances lysis of gram-positive microorganisms (5) and a dedicated step to eradicate non-DNA molecules (InhibitEX) that might interfere with the DNA extraction and downstream PCR (16). Cellular debris that is not removed can also result in a decreased yield and quality of DNA or RNA preparation (16).

The Semi-automatic easyMag® handles 24 fecal extractions simultaneously at approximately the same cost as the QIAamp DNA Stool Mini Kit in less time (40 minutes vs. 5 hours including pretreatment with TissueLyser, respectively) and obviously with much less hands-on time; therefore it is an attractive solution for laboratories handling many samples [13,15].

## Conclusions

In contrast to NanoDrop, quantification of DNA by the Qubit® system correlated with the density of the bands that appeared in electrophoresis gels for both whole genomic DNA and the PCR products. QIAamp Stool Mini Kit DNA extracts yielded higher amount of DNA and showed brighter bands on DGGE gels in comparison to DNA extracts obtained using easyMag®. However the semi-automatic easyMag® method is usable for molecular diagnostics, when DNA extracts are diluted 10 or 15 times.

Our study shows the necessity of testing different protocols for DNA extraction for samples of different origin in order to obtain optimal results and avoid false interpretations on the bacterial compositions and bacterial diversity.

## Availability and requirements

This study is based on laboratory work. No software or specific computer programs were used for the study.

## Abbreviations

Qiagen: QIAamp DNA stool mini kit; DGGE: Denaturing gradient gel electrophoresis; IBD: Inflammatory bowel disease; PCR: Polymerase chain reaction.

## Competing interests

The authors declare no competing financial interests.

## Authors’ contributions

KAK, AMP, HM, SP, CS participated in the design of the study. HM, AMP, SP and KAK drafted the manuscript. HM, LOBA were responsible for molecular genetic studies. All authors have read and approved the final manuscript.
